# Investigating revisit intention of medical tourists in China through nutritional knowledge, perceived medical quality, and trust in the physiologist: A recommendation on health tourism policy measures

**DOI:** 10.3389/fpubh.2022.893497

**Published:** 2022-08-26

**Authors:** Zhai Fengmin, Wu Baijun, Bai Jiangtao, Liu Li, Ataul Karim Patwary

**Affiliations:** ^1^Chengde Medical University, Chengde, China; ^2^School of Marxism Studies, North Minzu University, Yinchuan, China; ^3^Faculty of Hospitality, Tourism and Wellness, Universiti Malaysia Kelantan, Pengkalan Chepa, Malaysia

**Keywords:** revisit intention, medical tourists, nutritional knowledge, perceived medical quality, trust in the physiologist

## Abstract

Good medical care has long been a top priority in health tourism to keep the flow of visitors coming for medical treatment. Medical tourism encompasses a range of treatments, from basic check-ups to surgical operations. For its friendly character and high quality of service, China has earned a reputation as one of Asia's top destinations for health tourism. Along with India and Taiwan, Japan, Thailand, and South Korea are China's top tourism destinations. Considering the above fact, this study aims to examine the influence of nutritional knowledge, perceived medical quality, and trust in physiologists on revisiting the intention of medical tourists in China. This study is cross-sectional and follows a quantitative approach. The researchers used questionnaires as a survey tool to obtain information from the respondents. The respondents of this chosen international tourists in China who come for medical treatment purposes. A systematic random sampling technique was used to select the respondents, and 315 usable responses were collected from the respondents and proceeded with further analysis. The study conducted structural equation modeling using Smart PLS version 3. The results found that nutritional knowledge, perceived medical quality, and trust in physiologists significantly influence the revisit intention of medical tourists in China.

## Introduction

Chinese consumers with a substantial spending capacity are mostly interested in regular check-ups and cosmetic surgery. Many people go to South Korea for popular chemotherapy, cosmetic surgery, and skin procedures. Chinese patients accounted for 10% of the 320.000 tourists seeking treatment (1). Thailand is another popular location because of its high-quality treatments, affordable costs, and closeness to other famous vacation spots. Between January to April 2019, Chinese medical visitors grew by 30%. Aesthetic surgery, dental operations, and fertility treatments are some of the most in-demand services in the nation. China has several medical clinics that cater to international visitors. One of the most well-documented instances occurred on a tropical island in southern China. Traditional Chinese medical facilities established an international health centre that has since treated many foreign patients (2). Its services are in great demand.

Patients may get treatment at the facility while also enjoying Hainan's sun, beach, and surrounding natural beauty. The state government has made significant contributions to the growth of medical tourism. The government established an experimental zone for foreign medical tourism in a community on the island's east coast. It is referred to as the “Lecheng Pilot Zone” because it has medical products instruments from Japan, the European Union, and the United States that are currently unavailable in other regions of China. Provincial areas have promoted tourism projects and developed tourist routes like Beijing and Hainan since 2011.

The relationship between healthcare and tourist has resulted in one of the greatest service businesses in many nations. Medical tourism provides considerable economic advantages to many destination countries. Indeed, medical tourism is one of the world's fastest expanding tourist businesses (3, 4). To compete in an increasingly competitive medical tourism sector, many medical clinics in destination nations have upgraded their facilities and services to match those found in many top hotels (5, 6). These organizations often provide their overseas clientele with high-quality medical treatment and better service. Some potential difficulties or inconveniences overseas patient travelers encounter (e.g., language barriers, inefficient communication, low-quality medical care, uncomfortable atmospheres, low-quality services, unkind staff) are drastically decreased in dedicated medical tourism clinics (7, 8). To alleviate these difficulties and inconveniences in South Korea, many clinics have improved the quality of clinical services and lowered nurse-patient ratio and service performance (9, 10). As facilitators, these initiatives contribute to the rising number of overseas tourists visiting Korean clinics for medical treatment/healthcare/aesthetic treatments (11).

Medical tourism and allied enterprises have long been recognized as one of the most profitable hospitality industries in many destination nations, especially developing countries (12). The industry is growing at a breakneck pace, and rivalry in the worldwide medical tourism sector is heating up (13). In such a competitive climate, practitioners' primary objective is to acquire new medical travelers through marketing and motivate them to make repeat purchases *via* quality efforts/strategies (14). Recent statistics indicate that retaining current customers is around five times more lucrative than customer acquisition (15). Higher customer retention will likely boost any firm's profitability (16). Thus, in the medical tourism industry, identifying critical elements influencing medical tourists' repurchase decision-making procedures and comprehending their unique function is becoming more critical for every destination country and its associated medical facilities. Quality of product and service, contentment, and loyalty have long been viewed as essential factors in understanding the post-purchase behavior of customers. Most researchers think that these elements lead to positive attitudes about a business and impact loyalty and retention (17). Recognizing the significance of such elements, every organization in the tourism and tourist industries is becoming more concerned with efficiently controlling and enhancing quality, trust, and satisfaction. Thus, how to guarantee that consumers get a higher degree of quality from a product or service increase their level of pleasure and build their trust in the product's/performance services are some of the critical problems confronting today's hospitality and tourist marketers. This study examines the influence of nutritional knowledge, perceived medical quality, and trust in physiologists on revisiting the intention of medical tourists in China. This study is expected to provide insights for practitioners and academicians for further enhancement of practices in medical tourism and its research development.

## Literature review and hypothesis development

### Theoretical underpinning

The Theory of Planned Behavior (TPB) is aimed at improving the predictive power individual's intention. Initially, the behavioral intention was predicted by subjective norms and attitude; however, later on, Ajzen (18) realized that volition behavior control was excluded in the previous model. According to this theory, behavioral intention best predicts actual behavior. Behavioral intention has been described as a state of an individual's readiness to perform a certain behavior. This factor has also been identified to be an immediate antecedent of actual behavior (18). Several researchers have used the TPB to predict and understand people's intentions in various tourism contexts, such as leisure time physical activity (19), outdoor recreation (20–22), leisure travel behavior (23, 24), casino gambling (25, 26), and exercising behavior (27, 28). Most of the above studies demonstrated that the TPB could be used in predicting and explaining participation in diverse leisure activities or behaviors. Hrubes et al. (29) applied the TPB to predict and explain outdoor recreationists' hunting intentions and found several factors strongly influenced hunting intentions. The Theory of Planned Behavior underpins the framework established in this study as this study aimed to explain the revisit intention of tourists for medical purposes in China.

### Hypothesis development

#### Nutritional knowledge and revisit intention

Human growth, development, and long life depend on a healthy and productive diet. Healthy nutrition relies on adequate nutrients tailored to the individual's age, gender, and physiological state (30–33). A wide variety of dietary options are available to the public daily, making it difficult for individuals to get the required nutrients in adequate quantities. One food product is preferred over another because of the consumer's preferences. People's perception of basic tastes may be altered by biological changes, influencing their food choices. In addition, food preferences are influenced by psychological aspects that begin in infancy and continue throughout a person's life and diverse food-related learning experiences (34, 35).

The impact of food on healthy eating practices is well established (36). Dietary habits have been shown to reduce the risk of various chronic illnesses and death (37) WHO considers a lack of fruits, legumes, vegetables, nuts, and grains an unbalanced diet. A further risk factor for chronic illnesses is a diet high in fat, sugar, and salt (38, 39). When consumed in suitable quantities for developing and maintaining health *via* their vitamins and minerals, fruits and vegetables are considered healthy and savory (40). Studies show that individuals should take 400 g of fruit and vegetables daily, equivalent to five servings of 150–200 g each of veggies and 200 g each of fruit (41, 42). Fatty foods might be tasty, but they are also considered harmful depending on the dish in question. In order to maintain a healthy daily caloric intake, it is advised that individuals eat a maximum of 35 percent of their total daily calories from fat, which means avoiding meals high in saturated and trans-fatty acids (43). Proteins derived from animals, such as red and white meat and fish, eggs and dairy products, are likewise regarded as healthy and suggested for frequent consumption since they include important amino acids that promote growth and development (44–46). Consuming at least 48 g of whole grain food items with high carbohydrate content is also suggested since this increases satiety. It improves digestive health thanks to the dietary fiber they contain (47). Chocolate and ice cream are often delectable treats (48). This study posits the following hypothesis:

H1: Nutritional knowledge is positively related to revisit intention of medical tourists

##### Perceived medical quality and revisit intention

When it comes to describing what it means to judge a company's goods and services for excellence vs. alternatives given by rivals, existing literature on the subject offers few new conceptualizations (49). This quality has two primary aspects: core and service products (50). Core product quality measures how well a product performs concerning its price, while service product quality measures its performance due to interactions with the service staff (51). As part of the current research, perceived clinical quality refers to an individual's evaluations of the core medical product performance (e.g., excellence of medical care and clinical skills, wider access to medical products). In contrast, quality of service indicates the assessment of the performance of medical practitioners and staff (e.g., delivering services skills and competencies). Even though several conceptions of contentment have emerged over the last few decades, academics generally believe that individual contentment is an appraisal of the total experience with consuming (52). Customers' desire and ability to make more purchases will rise if they have a positive impression of their whole shopping experience (53).

Research has shown that quality and pleasure are key in creating intentions (54, 55). There is a clear link between quality and satisfaction, and the connection between quality and satisfaction is a substantial predictor of satisfaction (56, 57). The service links customer satisfaction and behavioral intentions, and product quality impacts customer satisfaction (58).

Li and Shang (59) found that in the service industry, the quality satisfaction connection is crucial to creating one's intention. These elements likewise influence customers' behavior (60, 61). Shen and Yahya (62) repeatedly found that the quality components of food, atmosphere and service had a substantial impact on satisfaction, and these linkages could explain the establishment of an intention. According to these most current research findings, the quality-satisfaction link heavily influences individuals' decision-making processes. This study posits the following hypothesis:

H2: Perceived medical quality is positively related to revisiting the intention of medical tourists

##### Trust in physiologists and revisit intention

The customer-provider relationship's long-term sustainability depends heavily on the mutual trust of both parties (63, 64). Ndubisi and Nataraajan (65) defined expectations formed by the customer that the provider is trustworthy and can be relied upon to execute its commitments. Academics widely accept trust as an efficient technique for reducing or exacerbating the causes of ambiguity (66, 67). Both employee/staff and company policies/practices must be trusted in order for trust to exist. One aspect of trust is dependent on client impressions of employee behavior/performances in a service experience context, while another aspect relies on corporate performance, such as its rules and procedures.

According to the idea of the agency, regardless of the depth of the connection between a firm and its clients, trust is more likely to lead to loyalty (68). Client business relation type was shown to have a moderating effect on intention development, and confidence in both the workers and firm was established based on customers' satisfaction with techniques used to manage complaints. In turn, this trust influenced customers' inclinations to promote good word-of-mouth and to repurchase. Sullivan and Kim (69) found that consumers' levels of trust strongly influenced their willingness to repurchase a product online. Customers' confidence in a seller grows when they have a positive experience buying from an online store. Customer satisfaction with exceptional product performance increases their faith in the provider's dependability and integrity, leading to the success of these satisfying experiences as a key influencer of repeat-purchase willingness. According to Gu et al. (70), trust is especially important in medical tourism because of the rising concerns about inadequate continuity of treatment and low-quality care in the quickly developing international medical business. Overall, this prior research has experimentally supported the idea that patient-satisfied customers are a substantial driving factor of trust and that this confidence plays an important part in creating behavioral intentions about medical treatment. This study posits the following hypothesis:

H3: Trust in physiologists is positively related to revisiting the intention of medical tourists.

## Methodology

### Sampling design and data collection

This study is cross-sectional and follows a quantitative approach to examine the influence of nutritional knowledge, perceived medical quality, and trust in physiologists on revisit intention of medical tourists in China. A cross-sectional study usually involves measuring all variables for all cases within a short period and once only. A systematic random sampling technique was used to select the respondents, and 315 usable responses were collected from the respondents and proceeded with further analysis. The researchers used questionnaires as a survey tool to obtain information from the respondents. The respondents of this chosen international tourists in China who come for medical treatment purposes.

### Measurement of the study

The measurement of this study was adopted from previous studies. One-to-seven-point Likert scales have been used to measure responses (1) Strongly disagree, (2) Disagree, (3) Somewhat disagree, (4) No opinion/ Neutral, (5) Somewhat agree, (6) Agree, (5) Strongly Agree. Five items were adopted from Oh (71) and Taylor and Baker (72) to measure perceived medical quality. Nutritional knowledge was measured using five items adapted from Ceylan et al. (73). Trust in physiologists was measured using five items adapted from Santos and Basso (74). Revisit intention was measured using three items adapted from Han (75).

### Data analysis

SEM measures and accommodates observed variables, representing any ambiguity in a construct of latent variables and simultaneously explaining casual relationships among latent and observed variables (61, 76). Besides, some of the measurement errors prevalent in tourism research can be solved using PLS-SEM with many latent variables (77–79). The initial analysis was conducted using SPSS version 23, and structural equation modeling was conducted using Smart PLS version 3.

## Analysis and hypotheses testing

The researchers examined the data using PLS-SEM to assess nutritional knowledge, perceived medical quality, revisit intention and trust in physiologists. We report results using a significance level at *p* < 0.01 and *p* < 0.001.

To develop the research variable, several important procedures were followed to construct validity, reliability, and content validity. In terms of content validity, a variety of operational aspects were evaluated using pre-existing research and multiple-item measurements. Both confirmatory and exploratory analyses confirm the factorability of the variables. An example of this is depicted in Figure 2. One item had a loading above 0.60, and the rest exceeded the recommended 0.70 loadings. Table 1 lists all the constructs, and the Cronbach alpha for each is ≥0.70.

**Table 1:** Construct validity and reliability.

**Variables**	**Cronbach's alpha**	**Composite reliability**	**Average variance extracted (AVE)**
Nutritional knowledge	0.896	0.922	0.703
Perceived medical quality	0.916	0.937	0.749
Revisit intention	0.881	0.918	0.738
Trust in physiologist	0.862	0.901	0.649

We checked the average variance extracted to ensure convergent validity and found that every variable was more significant than or equal to the recommended value of 0.50. As a result, all variables point to the validity and dependability of the content. Furthermore, the overall reliability of the composite was above the standard.

Figure 3 shows the factor loading of individual items and confirms the confirmatory factor analysis. To evaluate the discriminant validity of the 4-variables used in the study, Heterotrait-and Monotrait (HTMT) analysis was performed.

**Figure 1:**
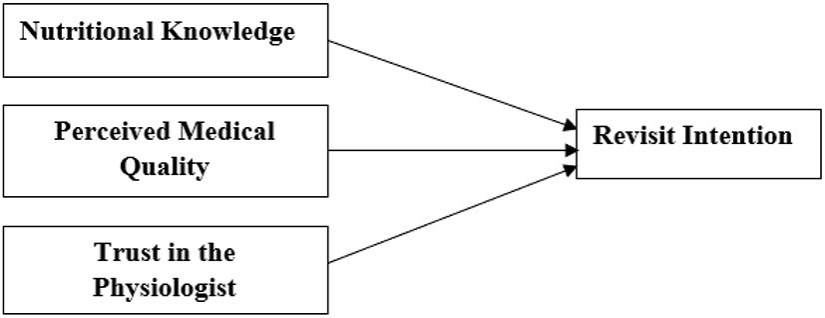
Research framework.

The result of the confirmatory factor analysis shown in Table 2 and Figure 1 supports the empirical evidence of the distinctiveness of the variables. No correlation exceeds the limit of maintaining an HTMT value of 0.85. Thus, all the study variables ensured the discriminant validity for further analysis.

**Table 2:** Discriminant validity (HTMT).

	**Nutritional knowledge**	**Perceived medical quality**	**Revisit intention**	**Trust in physiologist**
**Nutritional knowledge**
Perceived medical quality	0.123			
Revisit intention	0.234	0.337		
Trust in physiologist	0.152	0.367	0.369	

**Figure 2:**
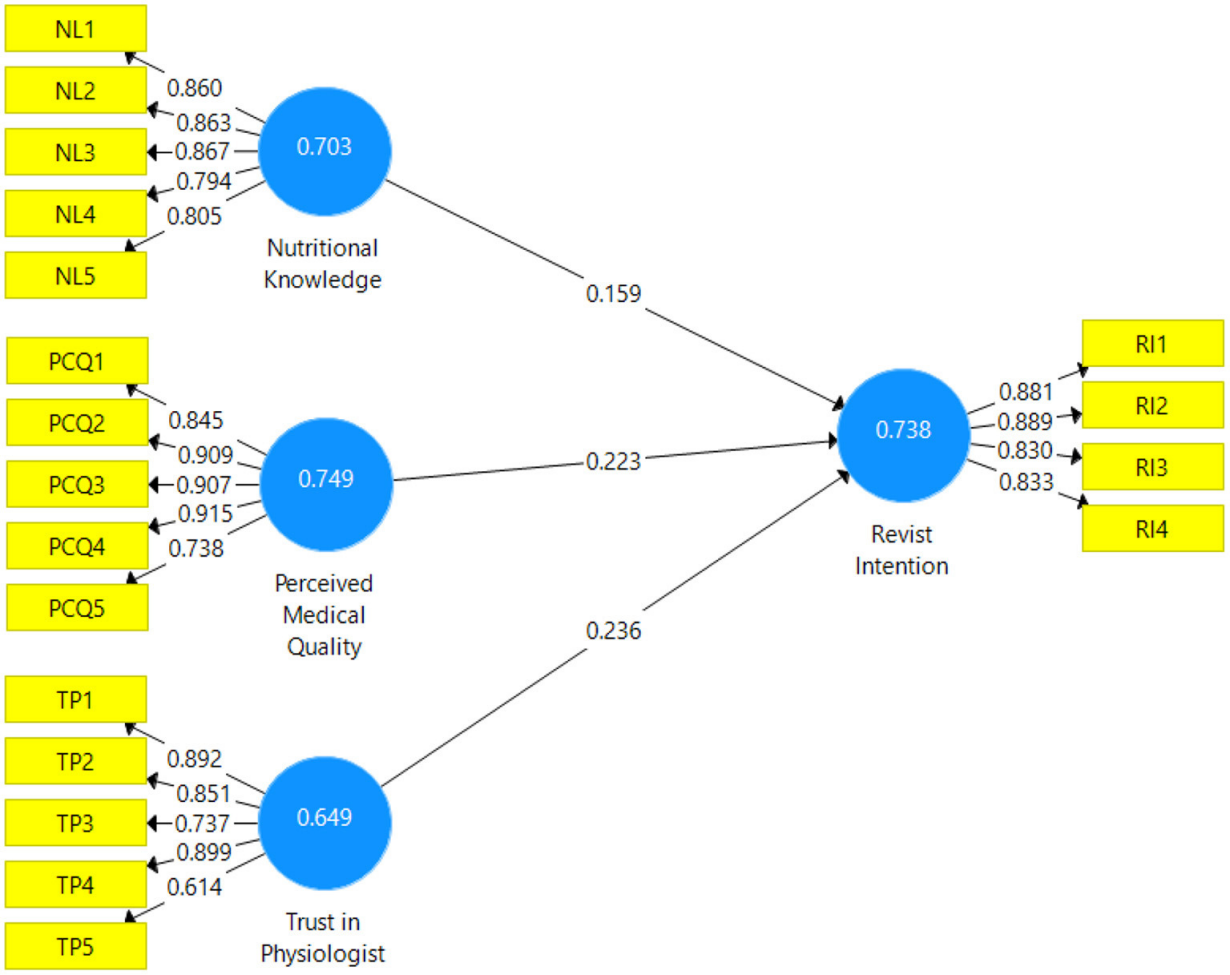
Measurement model.

**Figure 3:**
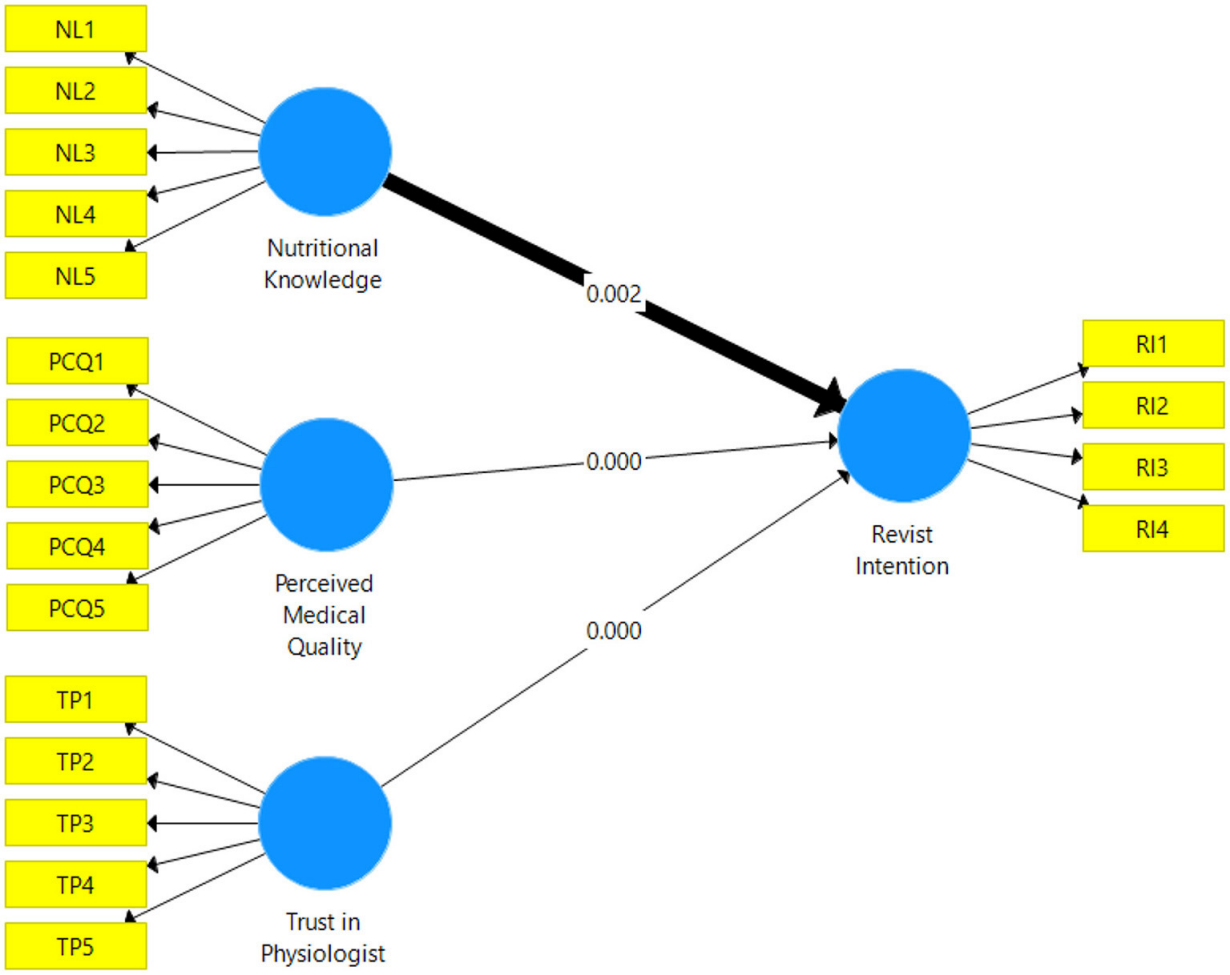
Structural model of the study.

### Structural equation modeling

In Smart-PLS, two models examine the effects of independent variables on the dependent variable: the measurement model and the structural model. These include constructing validity and reliability, which have already been discussed in detail. R square is also shown in the structural modeling equation for the ability of independent variables to predict the outcomes of their underlying dependent variables. The three variables chosen to have an R2 of 0.178, indicating a connection between nutritional knowledge, perceived medical quality, revisit intention and trust in physiologist. As a further measure of model fit, the SRMR (statistical correlation coefficient) was calculated, which was found to be 0.07.

As demonstrated in Table 3, the nutritional knowledge (β = 0.159, T value = 3.166, *P*-value = 0.002), perceived medical quality (β = 0.223, T value = 3.931, *P*-value = 0.000), and trust in physiologist (β = 0.236, *T* value = 4.263, *P*-value = 0.000) all have a significant influence on patient satisfaction. Nutritional knowledge has the greatest impact on revisit intention. Besides, perceived medical quality has a significant positive influence on revisit intention. And trust in physiologist has a positive effect on revisit intention, as does perceived value positively affect patient satisfaction. As a result, all hypotheses are supported.

**Table 3:** The direct effects of HRM practices on operational performance.

	**Original sample**	**Sample mean**	**Standard deviation**	**T Statistics**	***P*-Values**
	**(O)**	**(M)**	**(STDEV)**	**(|O/STDEV|)**	
Nutritional knowledge -> revisit intention	0.159	0.167	0.050	3.166	0.002
Perceived medical quality -> revisit intention	0.223	0.225	0.057	3.931	0.000
Trust in physiologist -> revisit intention	0.236	0.239	0.055	4.263	0.000

## Discussion and conclusion

Although medical tourism has grown rapidly, little is known about the significance of quality, satisfaction and trust in the post-purchase behavior of overseas patients. The major goal of this research was to build a conceptual background that clearly explains the creation of foreign medical tourists' intentions by examining the influence of perceived medical contentment, service quality, confidence in the personnel and clinic, and pricing reasonableness. Study participants were asked about their intentions to return to the clinic and the target country for medical treatment, and researchers looked at the relationships between these constructs to determine the moderating effect of perceived price rationality, as well as the relative importance of the various study variables that influence participants' intentions.

The current research effectively integrated the theoretical framework explaining the establishment of repurchase intention into the health center and its medical staff quality and trust. Medical travelers' opinions of price reasonableness were also included in this research, which effectively identified the underlying moderating effect of this variable. Although we do not claim that our conceptual model is highly robust, our model illustrated how international medical tourists' behavioral intentions might be developed. Although our theoretical framework incorporates these crucial elements, it has a solid foundation. Despite the lack of research on medical tourists' price-related decision-making in the medical tourism business, this study adds to that literature. To retain and grow their client base, medical clinics and the nations where they do business need to understand the critical role that pricing plays in attracting new business and maximizing existing income. Any transaction in which a client is engaged is likely to be judged on the basis of how they exploit it (80, 81). Customers are generally more inclined to think of a company's pricing as fair the more advantages they obtain (82, 83). Understanding the complexity of reasonable pricing is essential for practitioners who want to encourage repeat customers to believe they can expect a wide range of essential outcomes from their purchases [e.g., complementary aesthetic services to non-aesthetic repeaters (e.g., skincare evaluation, chemical peels for increasing skin glow, facial cleanses), gift certificates for local restaurants, local souvenirs]. Segmenting the market is also a good idea. Some developed countries choose lower-cost medical care, although upper-class citizens from developed and developing countries go overseas for high-quality and secure medical treatment while seeking hotel-style comforts in clinics rather than spending several hours in crowded areas (84). Customers of this kind will want detailed service and price strategies. Even though the costs of some treatments are exorbitant, wealthy patients who go to the clinic for treatment may think that these higher costs are justified since the clinic provides a luxurious amenities floor with hotel-style facilities and medical care.

### Policy implications

The study's policy ramifications are clear. Hospital use in urban and rural locations differs from a new routine due to evidence of rural inhabitants traveling to metropolitan areas for medical care. With the adoption of intra-provincial referral systems, branches of major hospitals in suburban regions have to be given greater attention than before. The central government should consider the local metropolitan community and the adjacent rural population when supplying precise medical resources. Facilities and resources should be distributed fairly and efficiently. Other developing nations with comparable medical systems and rapid urbanization may benefit from this study's findings. Because of this, policy implications may be used as a point of reference.

### Practical implications

This study examines the influence of nutritional knowledge, perceived medical quality, and trust in physiologists on revisiting the intention of medical tourists in China. This study is expected to provide insights for practitioners for further enhancement of practices in medical tourism and its research development. The outcome of this study is expected to benefit the practitioners by looking into the aspects of Chinese tourism industry, more specifically medical tourism. The practitioners will get more ideas on tourists' perspectives how it can be improved in future in terms of nutritional knowledge, perceived medical quality, and trust in the physiologist. The practitioners could take the note based on the study's outcomes and utilize it practically for more positive implications.

### Limitations and future directions of the study

Further studies should examine the variations in perceived and expected efficiency between non-profit and for-profit medical institutions, between private and public-owned facilities and between the perspectives of patients and service providers. Patients' expectations and satisfaction could also be analyzed to see statistical significance variations between persons in a healthcare facility due to an injury or recovery and those who utilize medical services to enhance their wellbeing. New items and questions on the significance of specific characteristics for patients might be added to the study instrument.

## Data availability statement

The raw data supporting the conclusions of this article will be made available by the authors, without undue reservation.

## Author contributions

ZF: conceptualizing and introduction. WB: literature review. BJ: method. LL: data collection and reporting. AP: data analysis. All authors contributed to the article and approved the submitted version.

## Conflict of interest

The authors declare that the research was conducted in the absence of any commercial or financial relationships that could be construed as a potential conflict of interest.

## Publisher's note

All claims expressed in this article are solely those of the authors and do not necessarily represent those of their affiliated organizations, or those of the publisher, the editors and the reviewers. Any product that may be evaluated in this article, or claim that may be made by its manufacturer, is not guaranteed or endorsed by the publisher.
